# Structural and Functional View of Polypharmacology

**DOI:** 10.1038/s41598-017-10012-x

**Published:** 2017-08-31

**Authors:** Aurelio Moya-García, Tolulope Adeyelu, Felix A. Kruger, Natalie L. Dawson, Jon G. Lees, John P. Overington, Christine Orengo, Juan A. G. Ranea

**Affiliations:** 1 University College London, Institute of Structural and Molecular Biology, London, UK; 2 European Molecular Laboratory – European Bioinformatics Institute, Hinxton, UK; 3 Department of Molecular Biology and Biochemistry, Universidad de Málaga, 29071 Málaga, Spain; 4 CIBER de Enfermedades Raras (CIBERER), 29071 Málaga, Spain; 5 BenevolentAI, Churchway 40, NW1 1LW London, UK; 6 Present Address: Department of Molecular Biology and Biochemistry, Universidad de Malaga, 29071, Málaga Spain, CIBER de Enfermedades Raras (CIBERER), 29071 Málaga, Spain; 7 Medicines Discovery Catapult, Mereside, Alderley Park, Alderley Edge, Cheshire SK10 4TG UK

## Abstract

Protein domains mediate drug-protein interactions and this principle can guide the design of multi-target drugs i.e. polypharmacology. In this study, we associate multi-target drugs with CATH functional families through the overrepresentation of targets of those drugs in CATH functional families. Thus, we identify CATH functional families that are currently enriched in drugs (druggable CATH functional families) and we use the network properties of these druggable protein families to analyse their association with drug side effects. Analysis of selected druggable CATH functional families, enriched in drug targets, show that relatives exhibit highly conserved drug binding sites. Furthermore, relatives within druggable CATH functional families occupy central positions in a human protein functional network, cluster together forming network neighbourhoods and are less likely to be within proteins associated with drug side effects. Our results demonstrate that CATH functional families can be used to identify drug-target interactions, opening a new research direction in target identification.

## Introduction

Systems Pharmacology emerged to address the potential limitations of viewing drug action from the perspective of a single target and has provided some rationale for the need for multi-target approaches in drug discovery^1–3^. In particular, this field provides a growing body of evidence against the “magic bullet”, i.e. a drug acting on one molecular target, affecting one biological process and thus effecting a cure with few other consequences. Many drugs bind to multiple targets and molecular targets are involved in multiple processes and perform multiple biological functions. Therefore, the term polypharmacology was coined to describe the ability of drugs to bind multiple molecular targets and thereby affect multiple biological processes^4–6^. Even though polypharmacology is still used to refer to the fact that most drugs bind to multiple proteins^7^, its meaning has shifted recently to reflect exploitation of this characteristic i.e. polypharmacology is now understood as the design or use of pharmaceutical agents to either simultaneously interact with multiple functionally related targets that act together, or to inhibit targets that differ functionally from the primary target of the drug to produce additional relevant effects—thus repurposing^2, 8, 9^ the drug for new effects.

Target identification is a crucial task in polypharmacology and it is important to identify synergistic combinations of targets^1, 10^. Most human targets are proteins that are composed of more than one domain^11, 12^, but we lack a unified definition of protein domains. In general terms, domains are compact and functional structural units that can be considered the evolutionary and structural building blocks of proteins. Since domains are units of structure^13^ and there is a limited repertoire of domain types^14^, they are combined to form different proteins with different overall functions^15^.

Recent studies have shown that protein domains mediate the interactions between a drug and its targets^16–18^. It has also been shown that domains are a major factor in the multi-target characteristics of approved and experimental drugs^19^, tend to contain binding sites^18^, and that there are privileged druggable protein domains^20^. Therefore, since a particular structural domain is likely to be the druggable entity in a protein target and since proteins have a modular structure and domains recur in different proteins, a reasonable explanation for the fact that a compound binds different protein targets is that they share a domain that is the actual target for the compound.

Under the accepted and general definition that a protein domain is a functional and structural module within a protein, there are several ways to identify and classify protein domains^21^: classification based on structure, SCOP^22^ and CATH^23^; classification based on sequence, Pfam^24^; and function oriented domain classifications such as the functional families classified in CATH, CATH-FunFams^23, 25^. CATH-FunFams group together relatives likely to have highly similar structures and functions^26^, and have been highly ranked in the International Critical Assessment of Functional Annotation^27, 28^.

In this work, we assess the pharmacology of CATH-FunFams and we explore their ability to mediate the interactions between drugs and their protein targets. We found that drug targets are overrepresented in some 81 druggable CATH-FunFams, whose relatives are structurally similar and contain conserved drug binding sites. These druggable CATH-FunFams group together in the protein functional network forming communities, and tend not to contain proteins associated with drug side effects. Therefore, druggable CATH-FunFams are enriched in potential drug targets. We propose that CATH-FunFams are a reasonable annotation level to study how drugs can interact with multiple targets, offering valuable insights for use in drug polypharmacology with potential applications in target identification and drug repurposing.

## Results and Discussion

### Drug binding proteins are found in a small set of CATH-FunFams

Protein domains are classified into protein domain superfamilies when they share a clear evolutionary relationship derived from similarities in their sequence, structure or both. In the CATH classification, a superfamily is sub-classified into functional families (CATH-FunFams) which group domains sharing significant structural and functional similarity. These groupings are achieved by clustering together relatives that have highly similar patterns of sequence conservation and likely specificity determining residues. CATH-FunFams have been benchmarked using experimentally characterised proteins in the Enzyme Classification (EC), SFLD and GO^29^ and have been independently validated by the CAFA independent assessment of function annotation^28^.

There have been a number of efforts to describe the portion of the genome susceptible to interact with drugs conceptualised by the term “druggable genome”, coined by Hopkins and Groom^20^. Despite the use of different definitions of protein families (SCOP, Pfam, InterPro) and their different estimates of druggable proteins reported, all the druggable genome studies noted that druggable proteins belong to certain protein families and therefore highlighted the existence of druggable domains^20, 30–32^. Therefore, the first step in analysing the role of CATH-FunFams in mediating drug-target interactions was the exploration of the druggable genome they depict.

**Figure 1:**
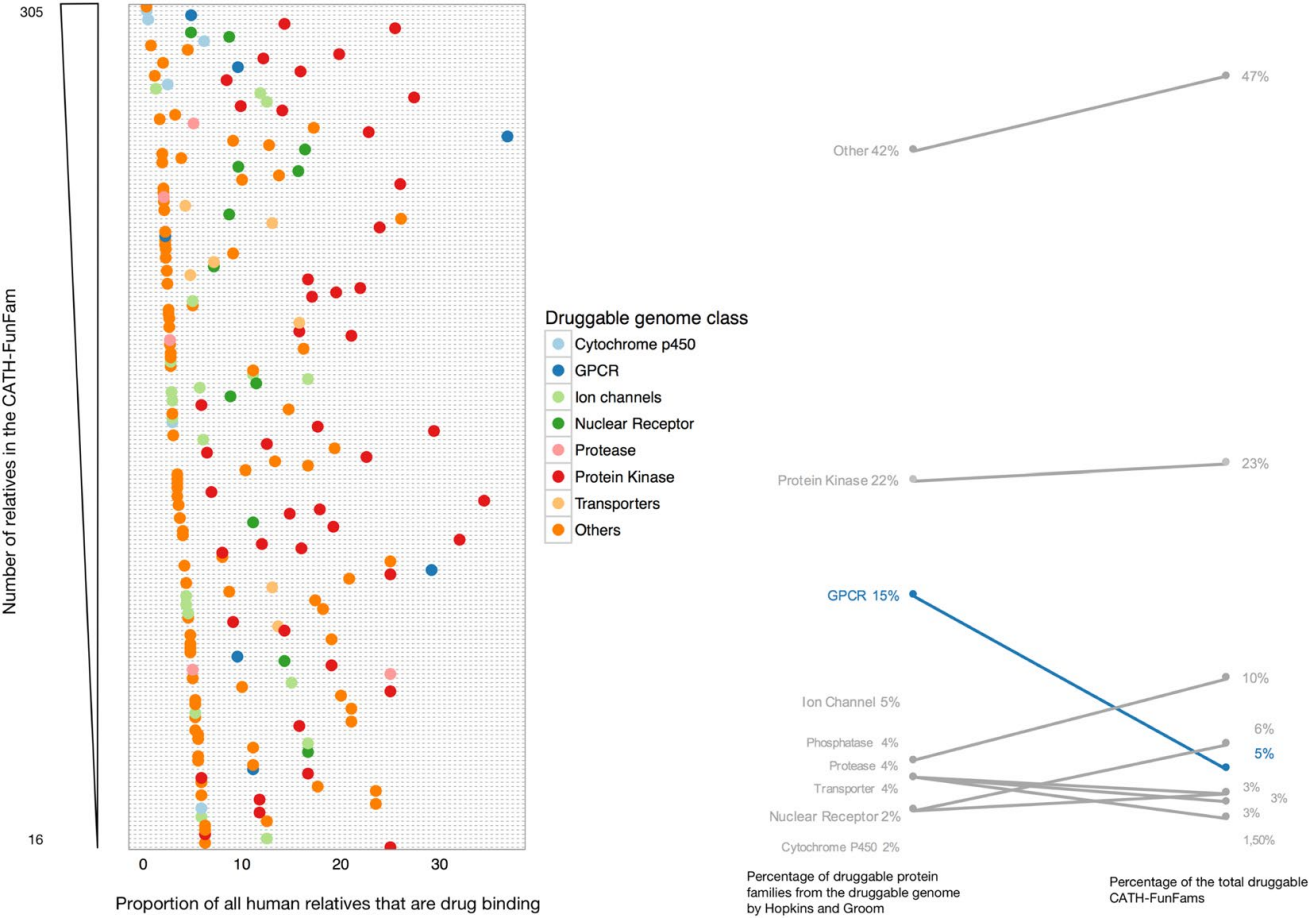
Proportion of drug-binding proteins in CATH-FunFams and the druggable genome. (**A**) Proportion of drug-binding proteins in 195 selected CATH-FunFams. FunFams were selected for having at least one drug-binding protein amongst their relatives and containing more than 2% of drug targets. (**B**) Slopegraph comparing the previous distribution of druggable protein families (i.e. the druggable genome) by Hopkins and Groom^20^ and our distribution of druggable CATH-FunFams.

There are 17229 CATH-FunFams containing 77082 human proteins. Most of them contain only a few protein relatives (the median number of relatives per CATH-FunFam is 3) but a few of them are very highly populated, such as the MHC class I antigen functional family (CATH-FunFam ID 3.30.500.10_3475) which has ca. 14% of the human proteins among its relatives. Using information in ChEMBL^33^, and based on their affinity to bind drugs, we identified a set of 787 human proteins capable of binding drugs (see Methods for details). This set of drug-binding proteins comprise drug targets (i.e. proteins able to bind approved drugs with high affinity) and drug off-targets (i.e. proteins that bind drugs at lower affinities). The drug-binding proteins are distributed in 875 CATH-FunFams (note that many proteins have more than one domain and therefore are represented as relatives of multiple CATH-FunFams). Most of these functional families are small, containing less than 2% of all human proteins. To gain a clear view of the druggable genome captured by the CATH-FunFams, we analysed 195 CATH-FunFams that have at least one drug-binding protein among their relatives and contain at least 2% of all the human proteins. Figure 1A shows the proportion of drug-binding proteins in these CATH-FunFams. Each FunFam point in the figure has been coloured according to major druggable protein class. Smaller functional families tend to have a higher proportion of drug-binding proteins, although for some druggable classes such as the protein kinases we find very large functional families with a high proportion of drug-binding proteins among their relatives. Figure 1B suggests that the CATH-FunFams capture well the previously reported druggable genome^20, 32^.

CATH-FunFams and other protein domain families are built using only protein structure and protein sequence data i.e. no drug or compound information is used to generate these protein classifications. Hopkins and Groom defined the druggable genome as a set of 130 InterPro protein families^20^ which were later expanded to an equivalent set of 182 Pfam domains^32^. Although there is no simple equivalence, we see that drug-binding proteins are found in in a few privileged CATH-FunFams, which describe the same major classes of druggable families as the Interpro and Pfam families of previous studies: Protein kinases, GPCRs and ion channels cover most of the druggable genome. A recent reassessment of the druggable genome identifies the same privileged druggable protein families, accounting for 44% of all human targets (GPCRs: 12%; ion channels: 19%; protein kinases: 10%; and nuclear receptors: 3%)^7^.

It is interesting to note that the number of GPCRs among the CATH-FunFams analysed is considerably lower than expected based on previous reports of the druggable protein families (see Fig. 1B). One reason for this lies in the difference in purity of functional annotations in CATH-FunFams, compared to annotations in other protein families. CATH-FunFams tend to separate domains according to their functional similarity and multi domain context, and therefore proteins assigned to a single InterPro or Pfam family will be split into several smaller CATH-FunFams. In fact, we find many GPCRs scattered across CATH-FunFams with few relatives, which reflects the diverse functionality of this target category. The lower proportion of GPCRs among the drug-binding CATH-FunFams is also explained by the structural nature of CATH functional families: GPCRs are membrane proteins many of which are structurally uncharacterised and therefore not classified in CATH yet, since CATH requires at least one relative with known structure to initiate a new domain superfamily. This limits the presence of GPCRs in CATH functional families.

### Identifying druggable CATH-FunFams—in which drug targets are significantly overrepresented

As mentioned already above, previous research has shown that drug binding sites are contained within protein domains^18^ and that protein domains mediate drug-target interactions^17^. Furthermore, the recurrent identification of domain families in the druggable genome suggests that the conserved sequence properties and functional similarities within a protein family, are associated with conservation of drug binding sites. This suggests that if one member of the protein family can bind a drug, other members would also be able to bind the same drug or a compound with similar physico-chemical properties^20^. Building on these ideas, we analysed the binding affinities of drugs that interact with multiple targets in CATH-FunFams to identify potential new targets.

We compiled a dataset of drug-targets and drug-off targets by querying ChEMBL for approved multi-target drugs and the human proteins to which they bind directly with high affinity. For each drug, we computed the statistically significant overrepresentation of their targets for each of the CATH functional families identified above in our survey of drug-binding CATH-FunFams (see above and Methods for details). Our resulting drug to CATH-FunFams mapping gave 359 statistically significant associations (Benjamini-Hochberg false discovery rate q-value < 0.001; see Supplementary Table S1) between 245 approved drugs and 81 CATH-FunFams, for which we will use the term druggable CATH-FunFams. We then investigated our druggable functional families to assess whether they have similar properties to protein drug targets and whether relatives in them are likely to bind drugs i.e. have putative drug binding sites.

### Similar drugs map to the same druggable CATH functional families

It is reasonable to expect that CATH-FunFams mediating the interaction between drugs and targets share the same characteristics as protein drug targets, studied by other researchers. One way to evaluate this is to test their compliance with the Similarity Property Principle (SPP), which establishes that drugs with similar molecular structure are likely to have the same properties^34^. Since the most relevant drug property is biological activity, produced by interaction with sets of targets^35^, to comply with the SPP a pair of drugs should have similar sets of targets. Therefore, we can compare protein drug targets and CATH-FunFams by evaluating the similarity in the sets of targets (interaction profiles) identified for structurally similar drugs.

**Figure 2:**
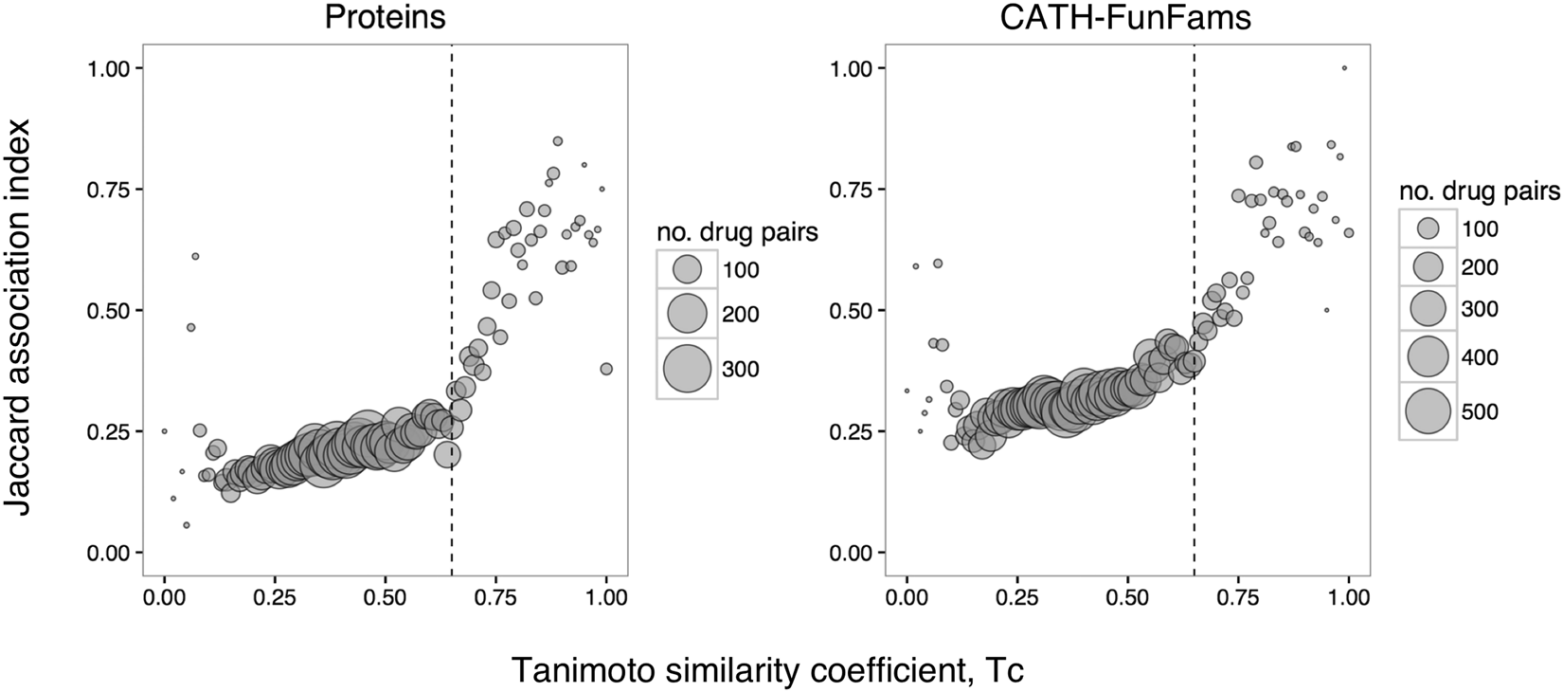
Correlation of the interactions profiles of a drug pair with their molecular similarity. Each circle is the average Jaccard index at a given bin of Tc similarity (bin size 0.01). The size of the circles is proportional to the number of drug pairs in the corresponding Tc bin. The vertical dashed line indicates the drug similarity threshold, Tc = 0.65 (see Supplementary Fig. S2).

Figure 2 shows the similarities of the interaction profiles of drugs as a function of their molecular similarity. For each drug, we determined two different interaction profiles: one is the set of protein targets it binds, and the other is the set of CATH-FunFams that contain the targets of the drug among their relatives. For each drug pair, we compute the similarity of their interaction profile by the Jaccard index. High values of the Jaccard index indicate that a pair of drugs have similar interaction profiles (either protein interaction profiles or CATH-FunFam interaction profiles). Thus, where the association index is 1 the two drugs have the same targets. We observe that for proteins, structurally similar drugs (Tc ≥ 0.65, see Supplementary Fig. S2) tend to have similar interaction profiles (i.e. they tend to bind the same targets) while structurally different drugs bind different targets. Furthermore, when we consider CATH-FunFams this observation is still apparent, suggesting that it is reasonable to map drugs to CATH-FunFams and identify druggable CATH functional families for target selection.

### Relatives in druggable CATH-FunFams are structurally similar with conserved binding sites

Protein targets contain drug-binding sites. Therefore, if our 81 druggable CATH-FunFams are enriched in new potential drug targets, their relatives must contain putative drug-binding sites. To evaluate the presence of drug binding sites in these 81 druggable CATH-FunFams we examined the 57 CATH-FunFams that have a crystal structure in the PDB, for their enrichment in druggable cavities, compared with a set of 100 random non-druggable CATH-FunFams 63 of which have a crystal structure. We found that 75% of the 57 druggable CATH-FunFams with structural information available, have cavities where binding of prodrugs or drug-like molecules is possible. Out of the set of 63 random non-druggable CATH-FunFams with defined structure, only 66% have cavities capable of binding drug-like molecules. Thus, druggable CATH-FunFams have a greater proportion of cavities able to bind drug-like molecules (p-val < 0.0001, Fisher exact test).

Furthermore, since CATH-FunFams have been shown to be structurally conserved if one or more relatives in a FunFam is known to bind a drug other relatives should share this property. That is, the 81 druggable CATH-FunFams should be enriched in potential drug targets ie contain relatives with the same drug binding properties.

**Figure 3:**
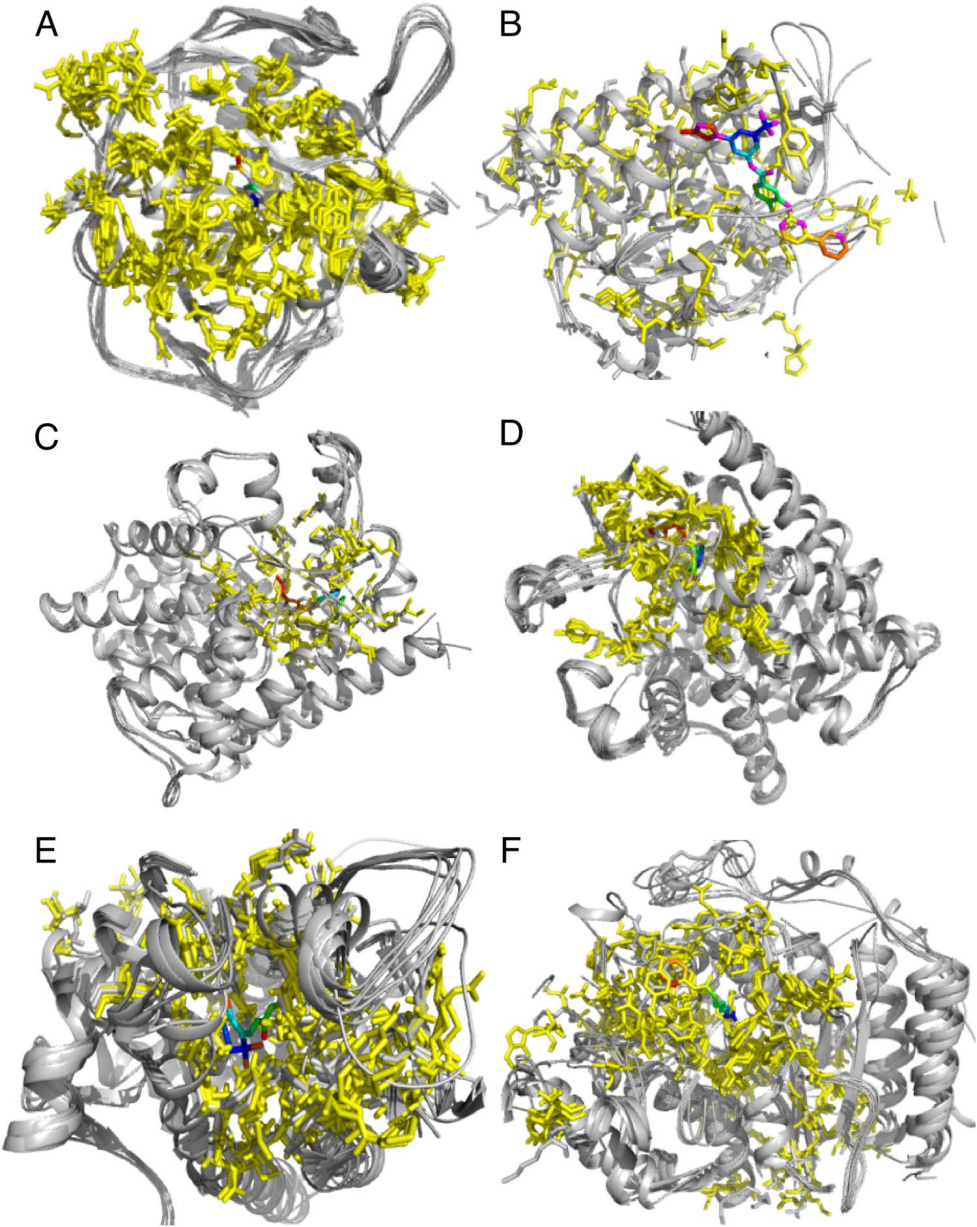
Conservation of the binding site within CATH-FunFams. Structural alignment of the CATH-Funfams associated with: (**A**) acetazolamide (CATH ID: 3.10.200.10-FF1430), (**B**) nilotinib (CATH ID: 1.10.510.10-FF78758), (**C**) Sildenafil (CATH ID: 1.10.510.10-FF78946), (**D**) tadalafil (CATH ID: 1.10.1300.10-FF1260), (**E**) Tretinoin (CATH ID: 1.10.565.10-FF5060) and (**F**) vorinostat (CATH ID: 3.40.800.20-FF2855) and the drug-target complexes of these drugs. In each case, the protein domain is grey, except the ligand binding residues, which have been coloured yellow. The drug molecules are coloured in rainbow.

We investigated this by analysing the drugs associated with CATH-FunFams for which there are structures of drug-target complexes in the PDB. Out of the 14 cases we found, we selected 6 examples to demonstrate the presence of similar drug binding sites in relatives within druggable CATH-FunFams (Fig. 3). It can be seen in Fig. 3 that the drug binding site is very well conserved amongst CATH-FunFam relatives, suggesting that all relatives of a CATH-FunFam associated with a drug, can bind that drug.

**Figure 4:**
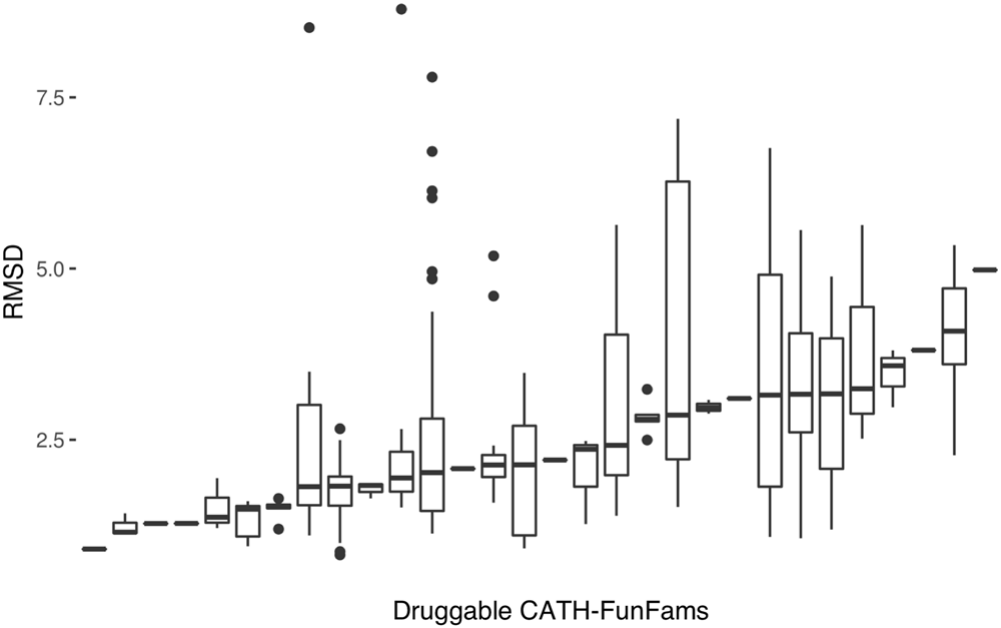
Normalised RMSD of druggable CATH-FunFams. Boxplots of the structural conservation within druggable CATH-FunFams. The RMSD is normalised by the number of residues in each structural alignment.

The mean RMSD for the aligned domain across all the six CATH-Funfams is 1.169 ± 0.812 Å suggesting that the druggable CATH-FunFams are structurally coherent. In order to evaluate the structural conservation of the druggable CATH-FunFams, we clustered the relatives within each druggable CATH-FunFam at 60% sequence identity and aligned the structural representatives of each cluster using the SSAP algorithm^36^. The median RMSD normalised by the number of aligned residues for the 30 druggable CATH-FunFams with structures available is below 5 Å. Furthermore, 83% of druggable FunFams with structure have median RMSD below 3 Å, see Fig. 4, implying that the druggable CATH-FunFams are indeed structurally conserved and that the high conservation of drug binding sites observed in the examples above can be extended to all the druggable CATH-FunFams.

### Network properties of drug targets and druggable CATH-FunFams

Although there are cases of drugs that bind with high affinity to multiple proteins differing from their primary targets, the most common scenario is that a drug binds with high affinity to its primary targets and with lower affinity to off-targets^7, 37^. This is a consequence of the obvious requirement to design selective drugs by increasing the affinity to the main target and decreasing drug affinity towards off-targets^38^. We have therefore hypothesised that the primary targets of a drug are proteins that bind the drug with high affinity and that off-targets are proteins that bind the drug with low affinity (see Methods).

We are also assuming that the binding of drugs with off-targets usually results in undesirable side effects^39, 40^. We therefore investigated the network properties of protein drug targets: their network centrality, and their ability to aggregate in the same regions of the network. We also investigated the connection between network properties of the protein drug targets and the side effects of the drugs. We hypothesised that protein drug targets that are dispersed in the human protein network might have be more strongly associated with side effects.

After examining the properties of protein targets, we repeated the analysis for relatives in druggable CATH-FunFams. We similarly hypothesised that druggable CATH-FunFams in which relatives are dispersed in the human protein network might have a higher association with side effects.

### Protein drug targets are central in the protein functional network

We first examined network properties of the 587 protein drug targets extracted from drug-proteins dataset (see Methods). As described above, drug targets are those proteins having high affinity for the drugs. Network centrality measures detect central nodes around which the network revolves. These central nodes correlate with the essential elements of the complex biological system described by that network^41, 42^. Protein drug targets have been shown to exhibit complex behaviour on molecular networks, both occupying central positions and connecting functional modules^43^.

Among the different measures of centrality, betweenness centrality best captures the ability of important nodes to be ‘between’ functional modules and also captures the link between the importance of a node in the network and the essentiality of the protein in the biological system^44^.

**Figure 5:**
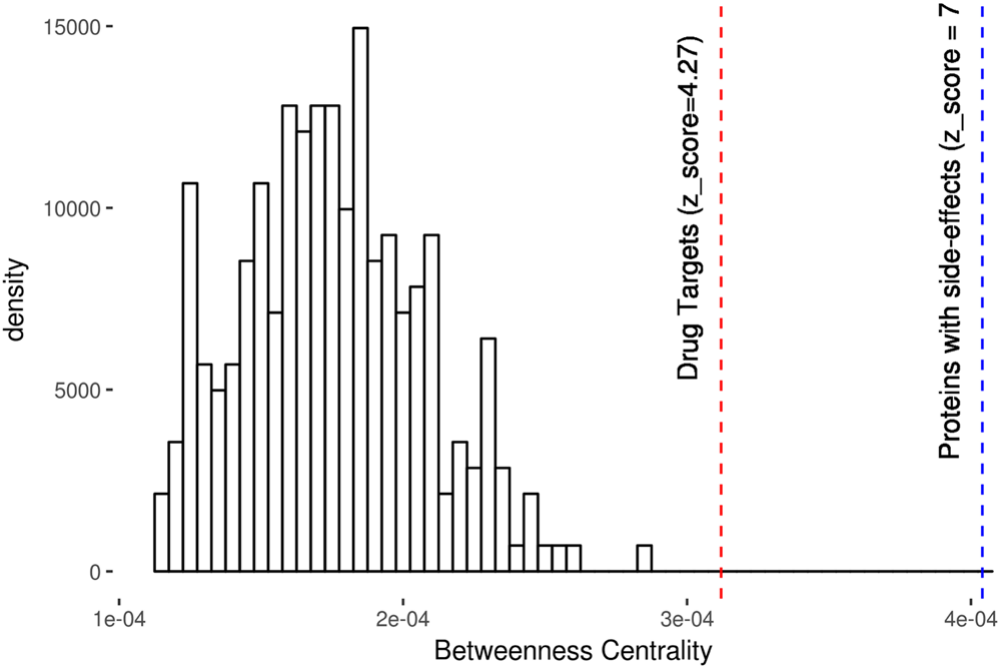
Betweenness centrality of drug targets. The mean betweenness centrality of drug targets (red line) and proteins associated with side effects in IntSide (blue line), in the protein functional network, is compared with the distribution of the mean betweenness centralities of random protein sets.

We analysed the betweenness centrality of protein drug targets using a network derived from functional associations between human proteins captured in the STRING database. STRING computes functional interactions between proteins through a combined score (ranging from 0 to 1), which indicates the confidence of a given association based on the different types of information supporting that association^45^. Figure 5 shows that drug targets have a higher betweenness centrality than proteins not associated with drugs—represented by sets of random proteins. However, some drug targets are associated with side effects and it can be seen in Fig. 5 that proteins with side effects (as identified by IntSide^46^ and including both drug targets and off-targets) are more central in the network.

We therefore observe that drug targets exhibit an interesting nuanced behaviour in the centrality-essentiality continuum. They are important elements in the protein functional network, often bridging two or more modules^43^, however, their essentiality is correlated with the presence of side effects^47^. That is, drug targets occupy central positions in the protein functional network, but if they are highly central (i.e. they are essential) targeting them produces side effects.

### Protein drug targets aggregate in the protein functional network forming neighbourhoods

Cellular functions are carried out by modules made up of interacting molecules^48^. Protein functional networks capture this phenomenon—where a link between two proteins means that both are involved in the same function or biological process—and are highly clustered, reflecting this modular design^49^. Therefore, proteins with similar functions tend to be connected or close to each other in the same neighbourhood of the protein functional network^50^. There are many examples of this phenomenon. For example: proteins associated with a disease tend to form modules^51^; modules in protein functional networks are used to predict and uncover protein functions inaccessible to experimental analysis^52, 53^; and proteins participating in the same signalling pathway form functional modules^54, 55^.

In order to measure the aggregation of the targets of each drug in the protein functional network, we used the STRING protein functional network to measure the network distance between the targets of a drug. We derived a similarity matrix from STRING where the value in row $i$, column $j$ had the STRING combined score between protein $i$ and protein $j$, i.e. the values of the similarity matrix indicate how well connected any two proteins are in the protein functional network. The mean similarity matrix value for a set of proteins is called the matrix similarity of this set, and represents how well connected these proteins are in the protein functional network. If a set of proteins has high matrix similarity the proteins will be strongly connected to each other in the protein functional network i.e. they will be in the same network neighbourhood.

**Figure 6:**
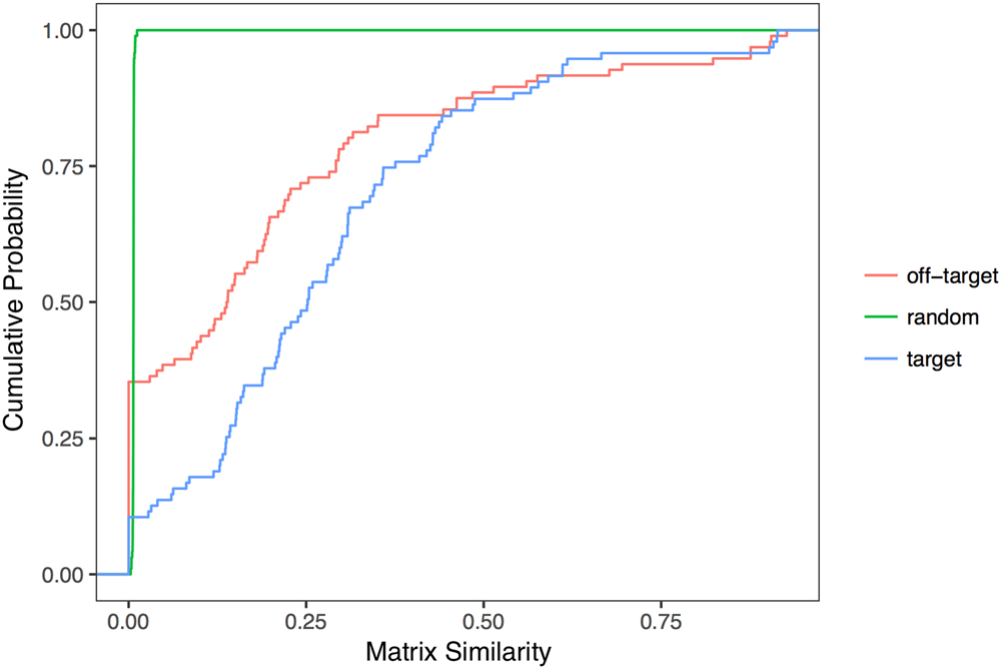
Drug neighbourhoods in the protein functional network. Cumulative distribution function of the matrix similarity of drug targets (blue line), off-targets (red line) and random sets of proteins (green line).

For each drug, we computed the matrix similarity of their protein targets, off-targets and a set of random proteins with the same number of proteins as the set of drug targets. Figure 6 shows the cumulative distribution function of the matrix similarities for these three datasets. Drug targets have higher matrix similarities than off-targets and both have higher matrix similarities than expected by chance. This means that drug targets tend to aggregate in the functional network, either forming modules of tightly connected proteins or by mapping closely to each other in the network; we dubbed this a drug neighbourhood. The tendency of targets to form drug neighbourhoods in the protein functional network is stronger than off-targets and remarkably larger than expected by chance. We also measured the ability of drug targets to form drug neighbourhoods using the network distance metrics developed by Menche *et al*.^51^ and proved, using this alternative approach, that drug targets tend to form drug neighbourhoods regardless of the method used to detect them (see Supplementary Fig. S4).

Thus, drug targets tend to cluster in the same neighbourhood of the functional network, whereas off-targets tend to disperse in the network. Since modules in the functional network imply proteins involved in the same process or biological function, we expect that the interaction between a drug and its targets will result in the alteration of one or a few biological functions (resulting in the drug’s pharmacological effect). By contrast the more dispersed nature of off-targets, which are more likely to be involved in disparate biological processes, will result in many side effects. In other words, proteins binding drugs with many side effects are likely to be more scattered in the functional network whereas proteins binding with less side effects will be more clustered in the functional network.

### Relatives of druggable CATH-FunFams are also central in the protein functional network and form drug neighbourhoods

**Figure 7:**
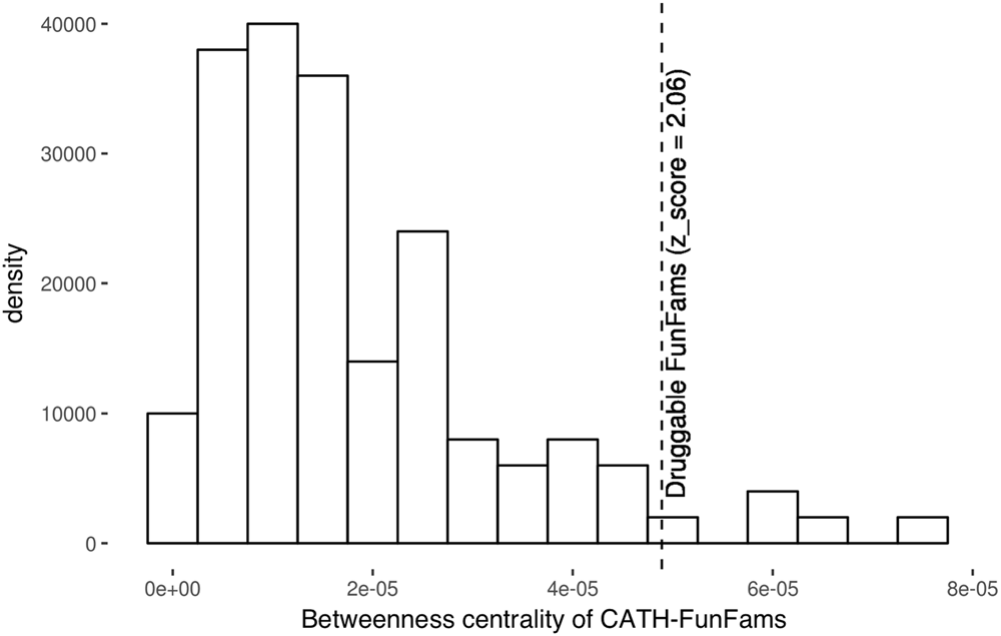
Betweenness centrality of druggable CATH functional families. The mean betweenness centrality of CATH-FunFams (dashed line) is compared with the distribution of the median betweenness centralities of random sets of non-druggable CATH-FunFams in the protein functional network.

We extended our network analysis to the relatives of druggable CATH-FunFams. As shown in Fig. 7 relatives of druggable CATH-FunFams are more likely to occupy central positions in the protein functional network than relatives from non-druggable CATH functional families, and whilst the relatives of druggable CATH-FunFams have betweenness centralities corresponding to drug targets, they don’t appear to be enriched in highly central (i.e. essential) proteins whose inhibition would lead to side effects.

The generally accepted idea is that relatives that are similar in sequence are likely to have similar interaction partners^56^ suggesting a correlation between sequence similarity of a protein pair and their closeness in the protein functional network. Since CATH-FunFams comprise sets of proteins with similar sequence characteristics, we would expect their relatives to be close in the network —regardless of whether we are analysing a druggable-FunFam or a non druggable-FunFam.

However, this is not what we observe; druggable CATH-FunFams are more likely to cluster in the same neighbourhood of the protein functional network forming drug neighbourhoods than CATH-FunFams that do not mediate the interactions between drugs and targets (median matrix similarities 0.54 and 0.23 respectively; Mann-Whitney-Wilcoxon test p-val < 0.01). This is in agreement with other research showing that the coupling between sequence distance and network distance is not simple. Superfamily relatives can have different interaction partners and would therefore not necessarily be close in the protein functional network^57^, and sequence divergent proteins often map close to each other on the protein interaction network^58^.

CATH-FunFams are designed to cluster relatives sharing similar functional determinants i.e. sequence patterns linked to function. However, our results show that this does not necessarily translate into proximity in the functional network. It is reasonable to suppose that CATH-FunFams whose relatives are more distributed in the network and which are therefore associated with side effects are more likely to comprise relatives that have more generic functions which can be exploited in multiple contexts i.e. diverse biological systems. These domains may have partner domains that are tuned to different sets of interactors, hence the distribution of the query domains into different parts of the protein network.

Nevertheless, our results show that the relatives of druggable CATH-FunFams are more likely to map together on the protein functional network. Presumably protein domains in these FunFams have been recurrently targeted in drug design because of their lower association with side effects.

### Calculating the likelihood that a CATH-FunFam is associated with side effects

To determine which CATH-FunFams are more likely to be associated with side effects, we built a logistic regression model of the probability of a CATH-FunFam being free of side effect proteins given its median matrix similarity. According to this statistical model, the probability that a CATH-FunFam which has its relatives completely dispersed on the functional network (i.e. matrix similarity = 0) does not contain proteins with side effects, is 31%. Whilst FunFams whose relatives have median matrix similarities greater than 0.48 have a probability higher than 50% that their relatives are not associated with side effects (p-val < 0.05). We can therefore be confident that the relatives of a CATH-FunFam with median matrix similarity above this threshold, i.e. relatives that cluster in the same neighbourhood of the functional network, are less likely to be associated with drug side effects.

### Drug neighbourhoods and druggable CATH-FunFams informs the safety of anticancer drugs

Anticancer drugs and protein kinase inhibitors in particular, constitute excellent examples of the advantages of studying drug target neighbourhoods and druggable CATH-FunFams to forecast drug side effects. The polypharmacology and variable side-effects of anticancer drugs provide an excellent case study to illustrate the use of our druggable CATH-FunFams in detecting potential interactions between drugs and proteins that lead to side effects.

Our drug-target dataset contains drugs belonging to up to 77 ATC level 2 categories. ATC L01, i.e. antineoplastic agents, is one of the most populated ATC categories in our dataset with 40 drugs. These cancer drugs are an important group comprising drugs that vary widely in the clustering of their targets in the protein functional network (matrix similarities range from 0.00 to 0.93) and the number of side effects (from 13 to 268 adverse drug reactions extracted from SIDER^59^).

Most of these antineoplastic drugs are protein kinase inhibitors (PKI) targeting the Protein Kinase superfamily. The inhibition of this superfamily by PKI and monoclonal antibody comprises the basis of targeted therapies in cancer^6, 7^. PKI have a great potential for polypharmacology; according to our data, PKI acts on a median number of 28 kinases with high affinity and just 3 of the 37 approved PKI (as of June 2016^7^) are specific to one kinase. Although PKI are considered less toxic than conventional chemotherapeutics, this is not always the case and their capability to affect multiple targets is considered to be a major cause of the observed side effects^6, 60^.

We observed a strong and significant negative correlation between the matrix similarity of proteins that bind cancer drugs and the number of side effects reported for these drugs in IntSide^46^ (Pearson’s correlation, r = −0.62; p-val < 0.01). The tendency of proteins that bind drugs with many side effects to be dispersed in the functional network holds when we analyse all the drugs in our datasets, although it is weakened by the lack of known side effects data for many of them. This suggests that drug neighbourhoods can provide information on likely drug safety and the drug’s potential to affect many biological processes via unintended interactions with other proteins.

We also observed a strong correlation between the scattering of relatives of CATH-FunFams on the protein functional network and the side effects of the PKIs associated with them (Pearson’s correlation, r = 0.58; p-val < 0.05); which agrees with our results that shows druggable CATH-FunFams forming drug neighbourhoods are likely to be enriched in potential targets.

When a kinase inhibitor is associated with relatives of a CATH-FunFam that form a tight drug neighbourhood we predict that the inhibitor will have few side effects, conversely a kinase inhibitor associated with a CATH-FunFam whose relatives are much more dispersed in the protein functional network is likely to have many side effects. This is exemplified by the contrast between lapatinib and erlotinib. Lapatinib is a tyrosine kinase inhibitor directed against the oncogenes EGFR and HER2 often used in breast cancer treatment^61^. Lapatinib is considered to be a well-tolerated cancer drug^62^, a characteristic that we could have derived from the association between lapatinib and the CATH-FunFam to which its targets belong and whose relatives form a tightly connected module in the network (see Supplementary Table S1). The median matrix similarity of this CATH-FunFam is 1. In contrast, we associated erlotinib—another EGFR inhibitor used in metastatic non-small cell lung cancer and pancreatic cancer, amongst other types of cancer, and implicated in many severe adverse drug reactions^63^—with a CATH-FunFam whose relatives are more dispersed on the functional protein network (median matrix similarity of 0.22). Therefore, we can anticipate the side effects caused by erlotinib through the network properties of the druggable CATH functional family associated with it.

Our drug to CATH-FunFams mapping also yields insights into the adverse side effects associated with sunitinib—a receptor tyrosine kinase inhibitor used in the treatment of renal cell carcinoma and other cancers^64^, which has raised safety concerns due to its many adverse reactions^65, 66^. We associated sunitinib with two CATH functional families, both very dispersed on the protein functional network (median matrix similarities of 0.16 and 0.15) and hence prone to contain relatives with many side effects. Thus, the broad polypharmacology of sunitinib, which is associated with functionally diverse targets is captured by our druggable CATH-FunFams. In other words, by targeting CATH-functional families which are highly spread on the protein functional network, sunatinib causes numerous side effects.

## Conclusion

We have provided fundamental support to the idea suggested by previous research that domain families, such as CATH functional families, mediate the interactions between drugs and their protein targets^16–19, 67, 68^. In this work, we conducted further analyses to test whether CATH-FunFams are druggable. We found that a small fraction of all CATH-FunFams are druggable and have shown using structural analyses that the domains in these families have the potential to be the druggable entities within drug targets.

The functional categories of CATH-FunFams agree with the functional categories of drug targets reported by other groups, based on druggable genome studies. The biased distribution of protein families in the druggable genome is also reflected in our druggable CATH-FunFams. The drug industry has relied upon targets that belong to a small number of protein families to develop new drugs, and this has produced a biased distribution of bioactivity data in ChEMBL, one of the principal data resources used in this research. The consequence of this bias in this study, is likely to be a limitation in the number of druggable CATH-FunFams that we identify. Even though we clearly identify druggable CATH-FunFams enriched in drug targets, there will be CATH-FunFams for which we cannot predict druggability because no drug information is available yet. Therefore, there may be more druggable FunFams than the 81 that we currently detect.

Druggable CATH-FunFams comply with the similarity property principle, that is drugs binding domains in these families show a correlation between similarity in their molecular structure and similarity in the targets to which they bind. Our studies also examined whether relatives within these functional families tended to be central in the protein functional network (i.e. have high betweenness centrality) and examined whether they were clustered together or dispersed in this network. These analyses revealed a tendency for relatives of druggable CATH-FunFams to be central, and that these relatives are likely to locate close together in the protein network forming drug neighbourhoods. The value of using CATH-FunFams as proxy targets is further enhanced by the fact that the extent of side effects associated with a drug can be gauged by the dispersion in the protein functional network of the relatives from the CATH-FunFam to which the targets of the drug belong.

In summary, our work supports the idea that drug protein interactions are mediated by drug-domain interactions. We have identified the CATH-FunFams as a reasonable annotation level for drug-target interactions that facilitates the identification of new drug targets, opening a new research direction in drug polypharmacology with potential applications in drug repurposing. Furthermore, we have demonstrated the high structural conservation of the CATH FunFam relatives which make them useful sets of proteins for drug repurposing, whilst analysis of their network properties can yield valuable information on likely side effects.

## Methods

### Drug-proteins dataset

We compiled a drug-protein dataset with 637 drugs and 679 human proteins (including drug targets and off-targets) by querying ChEMBL release 21. ChEMBL allows us to define drug target and drug off-targets based on the concentration at which a drug affects the protein. This provides a way to restrict our dataset to biologically meaningful drug-protein associations. We considered a drug as a small molecule with therapeutic application (THERAPEUTIC_FLAG = 1), not currently known to be a pro-drug, reporting a direct binding interaction with single protein (ASSAY_TYPE = ‘B’; RELATIONSHIP_TYPE = ‘D’; TARGET_TYPE = ‘SINGLE PROTEIN’), with a maximum phase of development reached for the compound of 4 (meaning an approved drug). For drug-target interactions we excluded non-specific interactions between small molecules and biological targets by filtering out weak activities (i.e. the activity of a drug against a human protein target should be stronger than 1 μM, where activity includes IC50, EC50, XC50, AC50, Ki, Kd; pchembl_value ≥ 6), while we used a pchembl value threshold between 1 and 4 to capture the less specific interactions between drugs and off-targets^33^.

### CATH-FunFams resource

We used CATH-FunFams v4.1 from CATH-Gene3D v12.0^25, 69^. CATH is a protein domain classification system that makes use of a combination of manual and automated structure- and sequence-based procedures to decompose proteins into their constituent domains and then classify these domains into homologous superfamilies (groups of domains that are related by evolution); domain regions in CATH are more clearly defined than in other domain resources by the use of structural data which is more highly conserved than the sequence. CATH-Gene3D is a large collection of CATH^23^ domain predictions for genome sequences ~20 million^70^. CATH superfamilies map to at least 60% of predicted domain sequences in completed genomes using in-house HMM protocols-and as high as 70–80% if more sophisticated threading-based protocols are used^71^. Domain sequences in each superfamily in CATH-Gene3D have been clustered into functionally coherent families (FunFams) using an in-house protocol^29^. This method identifies distinct FunFams within a superfamily having unique patterns of specificity determining residues. CATH-FunFams have been demonstrated to group together relatives likely to have similar structures and functions^29^. They have also been top-ranked in a blind test of functional annotation performance undertaken by the CAFA international assessment^28^. There are currently approximately ~100,000 CATH-FunFams identified amongst 2700 CATH superfamilies.

### Overrepresentation of drug targets in CATH functional families

We evaluated whether the targets ${\mathbb{T}}\,\{{{\rm{T}}}_{1},\ldots {{\rm{T}}}_{n}\}$ of a drug $d$ are significantly overrepresented among the relatives of a CATH-FunFam ${\mathbb{P}}\,\{{{\rm{P}}}_{1},\ldots {{\rm{P}}}_{n}\}$. In other words, we want to find whether the CATH-FunFam is enriched in the targets of *d*. For each combination of drug and CATH functional family we defined a test list as the relatives of the CATH-FunFam and a reference list containing all the drug targets. We also defined the expected value for the number of drug targets in the test list, as the number of drug targets that would be expected to be present in the test list based on the reference list. In other words, this is the expected probability that any drug target is a relative of a CATH-FunFam. For example, let’s assume there are a total of 1000 drug targets and 20 of them are relatives of the CATH-FunFam *FF*, then the expected value for *FF* is 0.02 i.e. 2%. If ${\mathbb{T}}$ contains 17 proteins we would expect that 0.34 of them are relatives of *FF*, if we observe more than 0.34 targets of *d* among the relatives of *FF*, the targets of $d$ are overrepresented on ${FF}$. Therefore, the overrepresentation of the targets of a drug among the relatives of a CATH-FunFam depends on the expected probability that a protein belongs to the CATH-FunFam. This probability is defined for each CATH-FunFam as the fraction of total drug targets that belongs to the CATH-FunFam.

We calculated a p-value (Benjamini–Hochberg corrected for multiple testing) to determine whether each observed overrepresentation is statistically significant by means of the binomial test. The binomial test evaluates the statistical significance of deviations from the binomial distribution of observations that fall into two categories: (i) the protein is a relative of the CATH-FunFam under consideration, or (ii) the protein is not a relative of the CATH-FunFam under consideration. The binomial distribution is the discrete probability distribution of the number of successes in a sequence of independent yes/no experiments each one with defined success probability. In our case the sequence of independent experiments is ${\mathbb{T}}$, the targets of $d$; a success is that a protein from ${\mathbb{T}}$ is a relative of the CATH-FunFam under evaluation. Each individual success has a probability ${P}_{{FF}}=\frac{{n}_{{FF}}}{N}$, which is the expected probability that a protein is a relative of ${FF}$, where ${n}_{{FF}}$ is the number of relatives of the CATH-FunFam ${FF}$ and $N$ is the total number of proteins relatives of all CATH-FunFams (i.e. all human proteins).

The null hypothesis is that the proteins in ${\mathbb{T}}$ are sampled from the same general population as the proteins in ${\mathbb{P}}$, and thus the probability of observing a target of $d$ as a relative of ${FF}$ is the same as observing any protein as a relative of ${FF}$ i.e. ${P}_{{FF}}$. Since we operate at a confidence level of 0.95, if p-value < 0.05 we reject the null hypothesis and we consider that the probability of observing the targets of *d* among the relatives of ${FF}$ is different from the probability of observing any set of proteins among the relatives of ${FF}$. Therefore, the p-value reported indicates if observing the targets of $d$ among the relative of ${FF}$ is likely to happen by chance. For p-values < 0.05 we consider the corresponding drug-CATH functional family association statistically significant and not likely to happen by chance. Note that the p-value is not the same as the binomial probability (not reported here), which is the probability that all the targets of $d$ are relatives of ${FF}$, when the probability of any target of $d$ is ${P}_{{FF}}$. The binomial test and all statistical computing in the following examples and in the rest of this work were performed with the R platform for statistical computing^72^.

For example, let’s consider ${FF}$ with ${\mathbb{P}}$ relatives and $d$ with ${\mathbb{T}}$ targets, then:

- Success: number of targets from ${\mathbb{T}}$ that are in ${\mathbb{P}}$
- Trials: number of targets of drug *d* in ${\mathbb{T}}$
- Probability of success under the null hypotesis: ${P}_{{FF}}=\frac{{n}_{{FF}}}{N}$
- Overrepresentation threshold: $$\mathrm{Trials}\times {P}_{{FF}}$$
- p-val: p-value of the binomial test (two-sided, confidence level = 0.95).

Tables 1 and 2 show two examples of overrepresentation of the targets of a drug across four CATH-FunFams, assuming there are 25 possible targets distributed amongst the CATH-FunFams. Example 1 illustrates the case of a drug with targets belonging mainly to one CATH-FunFam (Table 1), whereas in example 2 the drug’s targets are spread across several FunFams (Table 2). We observe in Table 1 that the targets of the drug are overrepresented for FF_2_ and FF_3_ but the overrepresentation is significant only in FF_3_; when the targets of a drug are spread across many CATH-FunFams (example in Table 2) we can see that the targets of the drug are overrepresented for FF_2_ but with no statistical significance.

**Table 1:** Example of the overrepresentation test for the case where a drug has 7 targets concentrated mainly in a CATH-FunFam.

FunFam	${{\boldsymbol{n}}}_{{\boldsymbol{FF}}}$	*P*_*FF*_	success	trials	Overrep. threshold	p-val
FF_1_	6	0.24	0	7	1.68	0.208
FF_2_	3	0.12	1	7	0.84	0.591
FF_3_	7	0.28	6	7	1.96	0.003
FF_4_	9	0.36	0	7	2.52	0.054

**Table 2:** Example of the overrepresentation test for the case where a drug has 5 targets distributed across several CATH-FunFams.

FunFam	${{\boldsymbol{n}}}_{{\boldsymbol{FF}}}$	*P*_*FF*_	success	trials	Overrep. threshold	p-val
FF_1_	6	0.24	1	5	1.2	1
FF_2_	3	0.12	2	5	0.6	0.113
FF_3_	7	0.28	1	5	1.4	1
FF_4_	9	0.36	1	5	1.8	0.66

### Molecular similarity calculation and pairwise associations of drug interaction profiles

We performed a significance analysis of the molecular similarity for our set of drugs, in order to choose a threshold Tc which will define a statistically significant level of similarity between any pair of drugs in our dataset. We retrieved the chemical table representing the chemical structure record of 2015 approved drugs (regardless of their targets) from ChEMBL release 21 and we obtained their MACCS molecular fingerprints^73^. We computed the Tanimoto similarity coefficients (Tc) between each drug and the remaining 2014 drugs using the RDKit package^74^. The Tc similarity quantifies the fraction of features common to the molecular fingerprints of the pair of drugs to the total number of features of the molecular fingerprints of each drug in the pair^75^. From these distributions of Tc values, we extracted the cumulative distribution function $F(t)$ that gives the probability of having a similarity less or equal than a given Tc value. A significance level (p-value) defined as $p=1{-}F(t)$ was assigned to every drug for each Tc value, according to Maggiora *et al*.^34^. Based on this analysis, the Tc threshold to define that two drugs have similar structure is 0.65 ($p$ = 0.005; see Supplementary Fig. S2).

For each drug, its interaction profile is the set of targets (proteins or CATH-FunFam domains) the drug is linked to. We analysed the interaction profile similarity between two drugs by means of the Jaccard association indices (${J}_{{ab}}$)^76^, defined as ${J}_{{ab}}=\frac{{n}_{a}\cap {n}_{b}}{{n}_{a}\cup {n}_{b}}$ where ${n}_{a}$ is the set of elements linked with drug $a$ (proteins or CATH-FunFam domains) and ${n}_{b}$ is the corresponding set of elements linked with drug $b$.

### Drug binding sites in CATH-FunFams

We used the Fpocket platform^77^ to detect druggable cavities in the structure of selected domains, i.e. cavities that can bind drug-like molecules. Fpocket is a fast protein pocket prediction algorithm that identifies cavities on the surface of proteins and ranks them according to their ability to bind drug-like small molecules. Thus, Fpocket assesses the ability of a given binding site to host drug-like organic molecules in terms of a druggability scoring function described in^78^.

To explore whether CATH-FunFams associated with drug binding consist of members with a similar binding pocket and similar amino acid residues, we looked in detail at six examples of FunFams which bind the drugs: acetazolamide, nilotinib, sildenafil, tadalafil, tretinoin and vorinostat. Structural domains from these four different CATH-FunFams were pairwise structurally aligned using SSAP^79^. SSAP scores were used to construct a distance matrix and maximum spanning tree which was then used to derive a multiple superposition of the structural relatives. Data on residues involved in binding each of the drugs of interest were extracted from the NCBI IBIS resource^80^ using the following PDB IDs as queries: 3ML5 for acetazolamide; 3CS9 for nilotinib; 1UDT for sildenafil; 1UDU for taladafil; 2LBD for treinoin; 4LXZ for vorinostat. These four PDB IDs were chosen as they were the only PDBs in each CATH-FunFam with drug binding information. These drug-binding residue positions were mapped onto the other structural domains using the SSAP alignment data. When producing the figures in PyMOL (www.pymol.org), the number of redundant structural domains in the acetazolamide and vorinostat alignments was reduced to improve clarity.

### Structural coherence of the druggable CATH-FunFams

The structural comparisons of relatives across the druggable CATH-FunFams were performed using the SSAP algorithm^79^. Since it is computationally expensive to compare all the relatives of each CATH-FunFam, we analysed the representatives of structural clusters within each CATH-FunFam. Relatives were clustered using CD-HIT^81^ at 60% sequence identity threshold, which indicates significant structural and functional similarity. Representative members of the clusters were used for all-against-all SSAP structural alignments, generating RMSD values normalised by the number of aligned residues in each case.

### Measuring protein neighbourhood in the functional network

We chose STRING to define the protein functional network because it is widely used and frequently updated. STRING compiles protein interaction and functional association data from several sources. These are benchmarked independently, and a combined score (which ranges from 0 to 1) is computed indicating the confidence of the association between two proteins. Therefore, protein associations have higher confidence when more than one type of information supports it^82^, the STRING data can be represented as a network based on the confidence of the functional associations of proteins: two proteins are linked in the network if the confidence of their functional association passes a established threshold. We kept high confidence protein associations (i.e. we applied a cut-off of 0.8 on the combined score) to model the protein functional network, which we used for the network centrality analysis in this work.

We transformed the STRING network (all edge weights) into a similarity matrix, by taking its adjacency matrix. The adjacency matrix of the full STRING network (i.e. no combined score cut-off) contains all the information of the functional associations between proteins: the value in row $i$, column $j$ had the STRING combined score (0–1) between protein $i$ and protein $j$. This adjacency matrix has the properties of a similarity matrix and reflects the integration of the disparate protein interaction types and sources implemented in STRING (see refs 53 and 83 for details on the use of matrices in data integration). Based on this matrix we defined the matrix similarity of a group of proteins as their mean STRING combined score, which reflects the closeness of these proteins in the protein functional network, i.e. proteins with high matrix similarity gather together in the protein functional network.

All data processing, statistics analysis and results plots were produced using Python and Networkx^84^, the R computing environment^72^, and the R library ggplot2^85^.

## Electronic supplementary material

Supplementary Information

Dataset 1

## References

[1] Berg EL (2014). Systems biology in drug discovery and development. Drug Discov Today.

[2] Anighoro, A., Bajorath, J. & Rastelli, G. Polypharmacology: Challenges and Opportunities in Drug Discovery. *J Med Chem*, doi:10.1021/jm5006463 (2014). 10.1021/jm5006463 24946140

[3] Moya-García AA, Morilla I, Ranea JAG (2014). Oncogenic Signalling Networks and Polypharmacology as Paradigms to Cope with Cancer Heterogeneity. Current Proteomics.

[4] Roth BL, Sheffler DJ, Kroeze WK (2004). Magic shotguns versus magic bullets: selectively non-selective drugs for mood disorders and schizophrenia. Nat Rev Drug Discov.

[5] Mestres J, Gregori-Puigjané E, Valverde S, Solé RV (2008). Data completeness–the Achilles heel of drug-target networks. Nat Biotechnol.

[6] Knight ZA, Lin H, Shokat KM (2010). Targeting the cancer kinome through polypharmacology. Nat. Rev. Cancer.

[7] Santos, R. *et al*. A comprehensive map of molecular drug targets. *Nat Rev Drug Discov* 1–16, doi:10.1038/nrd.2016.230 (2016). 10.1038/nrd.2016.230 PMC6314433 27910877

[8] Antolín, A. A., Workman, P., Mestres, J. & Al-Lazikani, B. Polypharmacology in Precision Oncology: Current Applications and Future Prospects. *Curr Pharm Des*, doi:10.2174/1381612822666160923 (2016). 10.2174/1381612822666160923115828 PMC5403974 27669965

[9] Chaudhari R, Tan Z, Huang B, Zhang S (2017). Computational polypharmacology: a new paradigm for drug discovery. Expert Opin Drug Discov.

[10] Lavecchia A, Cerchia C (2016). In silico methods to address polypharmacology: current status, applications and future perspectives. Drug Discov Today.

[11] Chothia C, Gough J, Vogel C, Teichmann SA (2003). Evolution of the protein repertoire. Science.

[12] Apic G, Gough J, Teichmann SA (2001). An insight into domain combinations. Bioinformatics.

[13] Orengo CA (1997). CATH–a hierarchic classification of protein domain. structures. Structure/Folding and Design.

[14] Wolf YI, Grishin NV, Koonin EV (2000). Estimating the number of protein folds and families from complete genome data. J Mol Biol.

[15] Kummerfeld SK, Teichmann SA (2009). Protein domain organisation: adding order. BMC Bioinformatics.

[16] Yamanishi, Y., Pauwels, E., Saigo, H. & Stoven, V. Extracting Sets of Chemical Substructures and Protein Domains Governing Drug-Target Interactions. *J Chem Inf Model*, doi:10.1021/ci100476q (2011). 10.1021/ci100476q 21506615

[17] Wang X (2012). Three-dimensional reconstruction of protein networks provides insight into human genetic disease. Nat Biotechnol.

[18] Kruger FA, Rostom R, Overington JP (2012). Mapping small molecule binding data to structural domains. BMC Bioinformatics.

[19] Moya-García AA, Ranea JAG (2013). Insights into polypharmacology from drug-domain associations. Bioinformatics.

[20] Hopkins AL, Groom CR (2002). The druggable genome. Nat Rev Drug Discov.

[21] Koonin EV, Wolf YI, Karev GP (2002). The structure of the protein universe and genome evolution. Nature.

[22] Murzin AG, Brenner SE, Hubbard T, Chothia C (1995). SCOP: a structural classification of proteins database for the investigation of sequences and structures. J Mol Biol.

[23] Sillitoe I (2015). CATH: comprehensive structural and functional annotations for genome sequences. Nucleic Acids Res.

[24] Finn RD (2014). Pfam: the protein families database. Nucleic Acids Res.

[25] Rentzsch R, Orengo CA (2013). Protein function prediction using domain families. BMC Bioinformatics.

[26] Dessailly BH, Dawson NL, Mizuguchi K, Orengo CA (2013). Functional site plasticity in domain superfamilies..

[27] Radivojac P (2013). A large-scale evaluation of computational protein function prediction. Nat. Methods.

[28] Jiang Y (2016). An expanded evaluation of protein function prediction methods shows an improvement in accuracy. Genome Biol..

[29] Das S (2015). Functional classification of CATH superfamilies: a domain-based approach for protein function annotation. Bioinformatics.

[30] Rask-Andersen M, Masuram S, Schiöth HB (2014). The Druggable Genome: Evaluation of Drug Targets in Clinical Trials Suggests Major Shifts in Molecular Class and Indication. Annu. Rev. Pharmacol. Toxicol..

[31] Overington JP, Al-Lazikani B, Hopkins AL (2006). How many drug targets are there?. Nat Rev Drug Discov.

[32] Russ AP, Lampel S (2005). The druggable genome: an update. Drug Discovery Today.

[33] Bento AP (2014). The ChEMBL bioactivity database: an update. Nucleic Acids Res.

[34] Maggiora G, Vogt M, Stumpfe D, Bajorath J (2014). Molecular similarity in medicinal chemistry. J Med Chem.

[35] Petrone PM (2012). Rethinking molecular similarity: comparing compounds on the basis of biological activity. ACS Chem. Biol..

[36] Taylor WR, Orengo CA (1989). Protein structure alignment. Journal of molecular biology.

[37] Csermely P, Agoston V, Pongor S (2005). The efficiency of multi-target drugs: the network approach might help drug design. Trends in Pharmacological Sciences.

[38] Kawasaki Y, Freire E (2011). Finding a better path to drug selectivity. Drug Discov Today.

[39] Duran-Frigola M, Aloy P (2013). Analysis of chemical and biological features yields mechanistic insights into drug side effects. Chemistry & Biology.

[40] Lynch JJ, Van Vleet TR, Mittelstadt SW, Blomme EAG (2017). Potential functional and pathological side effects related to off-target pharmacological activity. J Pharmacol Toxicol Methods.

[41] Jeong H, Mason SP, Barabási A-L, Oltvai ZN (2001). Lethality and centrality in protein networks. Nature.

[42] Jalili M (2016). Evolution of Centrality Measurements for the Detection of Essential Proteins in Biological Networks. Front Physiol.

[43] Csermely P, Korcsmáros T, Kiss HJM, London G, Nussinov R (2013). Structure and dynamics of molecular networks: A novel paradigm of drug discovery: A comprehensive review. Pharmacol. Ther..

[44] Yu H, Kim PM, Sprecher E, Trifonov V, Gerstein M (2007). The Importance of Bottlenecks in Protein Networks: Correlation with Gene Essentiality and Expression Dynamics. PLoS Comput Biol.

[45] Szklarczyk D (2015). STRING v10: protein-protein interaction networks, integrated over the tree of life. Nucleic Acids Res.

[46] Juan-Blanco T, Duran-Frigola M, Aloy P (2015). IntSide: a web server for the chemical and biological examination of drug side effects. Bioinformatics.

[47] Wang X, Thijssen B, Yu H (2013). Target essentiality and centrality characterize drug side effects. PLoS Comput Biol.

[48] Hartwell LH, Hopfield JJ, Leibler S, Murray AW (1999). From molecular to modular cell biology. Nature.

[49] Winterbach W, Van Mieghem P, Reinders M, Wang H, de Ridder D (2013). Topology of molecular interaction networks. BMC Systems Biology.

[50] Sharan R, Ulitsky I, Shamir R (2007). Network-based prediction of protein function. Mol Syst Biol.

[51] Menche J (2015). Uncovering disease-disease relationships through the incomplete interactome. Science.

[52] Ranea JAG (2010). Finding the ‘dark matter’ in human and yeast protein network prediction and modelling. PLoS Comput Biol.

[53] Hériché J-K (2014). Integration of biological data by kernels on graph nodes allows prediction of new genes involved in mitotic chromosome condensation. Molecular Biology of the Cell.

[54] Bhalla, U. S. & Iyengar, R. Functional modules in biological signalling networks. *Novartis Found. Symp*. **239**, 4–13– discussion 13–5–45–51 (2001). 10.1002/0470846674.ch2 11529315

[55] Cerami E, Demir E, Schultz N, Taylor BS, Sander C (2010). Automated Network Analysis Identifies Core Pathways in Glioblastoma. PLoS ONE.

[56] Sun MGF, Kim PM (2011). Evolution of biological interaction networks: from models to real data. Genome Biol..

[57] Reid AJ, Ranea JAG, Clegg AB, Orengo CA (2010). CODA: accurate detection of functional associations between proteins in eukaryotic genomes using domain fusion. PLoS ONE.

[58] Bueno A (2016). Exploring the interactions of the RAS family in the human protein network and their potential implications in RAS-directed therapies. Oncotarget.

[59] Kuhn M, Letunic I, Jensen LJ, Bork P (2016). The SIDER database of drugs and side effects. Nucleic Acids Res.

[60] Park SR, Davis M, Doroshow JH, Kummar S (2013). Safety and feasibility of targeted agent combinations in solid tumours. Nature Reviews Clinical Oncology.

[61] Burris HA (2004). Dual kinase inhibition in the treatment of breast cancer: initial experience with the EGFR/ErbB-2 inhibitor lapatinib. The Oncologist.

[62] Higa GM, Abraham J (2007). Lapatinib in the treatment of breast cancer. Expert Rev Anticancer Ther.

[63] Becker A, van Wijk A, Smit EF, Postmus PE (2010). Side-effects of long-term administration of erlotinib in patients with non-small cell lung cancer. J Thorac Oncol.

[64] Theou-Anton N, Faivre S, Dreyer C, Raymond E (2009). Benefit-risk assessment of sunitinib in gastrointestinal stromal tumours and renal cancer. Drug Saf.

[65] Amemiya T (2015). Elucidation of the molecular mechanisms underlying adverse reactions associated with a kinase inhibitor using systems toxicology. npj Syst. Biol. Appl..

[66] Carlisle B (2015). Benefit, Risk, and Outcomes in Drug Development: A Systematic Review of Sunitinib. JNCI Journal of the National Cancer Institute.

[67] Kruger, F. A., Gaulton, A., Nowotka, M. & Overington, J. P. PPDMs-a resource for mapping small molecule bioactivities from ChEMBL to Pfam-A protein domains. *Bioinformatics*, doi:10.1093/bioinformatics/btu711 (2014). 10.1093/bioinformatics/btu711 PMC4341065 25348214

[68] Pardo EP, Godzik A (2015). Analysis of individual protein regions provides novel insights on cancer pharmacogenomics. PLoS Comput Biol.

[69] Lee DA, Rentzsch R, Orengo C (2010). GeMMA: functional subfamily classification within superfamilies of predicted protein structural domains. Nucleic Acids Res.

[70] Lees J, Yeats C, Redfern O, Clegg A, Orengo C (2010). Gene3D: merging structure and function for a Thousand genomes. Nucleic Acids Research.

[71] Lees J (2012). Gene3D: a domain-based resource for comparative genomics, functional annotation and protein network analysis. Nucleic Acids Res.

[72] R C, Team R C. R: A Language and Environment for Statistical Computing. R Core Team. R Foundation for Statistical Computing (2014).

[73] Rogers D, Hahn M (2010). Extended-connectivity fingerprints. J Chem Inf Model.

[74] RDKit: Cheminformatics and Machine Learning Software. *RDKit: Cheminformatics and Machine Learning Software* Available at: http://www.rdkit.org. (Accessed: 30 June 2017)

[75] Willett P, Barnard JM, Downs GM (1998). Chemical Similarity Searching. J Chem Inf Model.

[76] Fuxman Bass JI (2013). Using networks to measure similarity between genes: association index selection. Nat. Methods.

[77] Le Guilloux V, Schmidtke P, Tuffery P (2009). Fpocket: an open source platform for ligand pocket detection. BMC Bioinformatics.

[78] Schmidtke P, Barril X (2010). Understanding and predicting druggability. A high-throughput method for detection of drug binding sites. J Med Chem.

[79] Orengo CA, Taylor WR (1996). SSAP: sequential structure alignment program for protein structure comparison. Meth Enzymol.

[80] Shoemaker BA (2012). IBIS (Inferred Biomolecular Interaction Server) reports, predicts and integrates multiple types of conserved interactions for proteins. Nucleic Acids Res.

[81] Li W, Godzik A (2006). Cd-hit: a fast program for clustering and comparing large sets of protein or nucleotide sequences. Bioinformatics.

[82] Szklarczyk D (2011). The STRING database in 2011: functional interaction networks of proteins, globally integrated and scored. Nucleic Acids Res.

[83] Erasmus JC (2016). Defining functional interactions during biogenesis of epithelial junctions. Nature Communications.

[84] Hagberg, A., Swart, P. & Chult, S. D. Exploring network structure, dynamics, and function using networkx (2008).

[85] Wickham, H. *ggplot2: Elegant Graphics for Data Analysis*. (Springer Science & Business Media, 2009).

