# Supplementary material for: Structural and Functional View of Polypharmacology
Source: Sci Rep. 2017 Aug 31;7:10102. doi: 10.1038/s41598-017-10012-x (PMC5579063; doi:10.1038/s41598-017-10012-x)
Supplement: Supplementary file 1 — Supplementary Information [file 41598_2017_10012_MOESM1_ESM.pdf]

Supplementary Information to Structural and Functional View of Polypharmacology

Aurelio Moya-Garcia, Tolulope Adeyelu, Felix A Kruger, Natalie L. Dawson, Jon G. Lees, John P. Overington, Christine Orengo and Juan A.G. Ranea

Supplementary Table S1. Drug to CATH-FunFam mapping. The names and codes of our association between drugs and CATH-FunFams are provided in a separate excel file. Druggable Genome class: type of protein family according to the categories of the druggable genome by Hopkins and Groom[1]. Number of Side Effects: Number of preferred MedDRA terms associated with the drug in the SIDER database[2]. Kernel Similarity: mean STRING combined score of the relatives of the CATH-FunFam. Probability of SE free: Probability that the CATH-FunFam do not contain a relative associated with side effects according to our logistic regression model (see main text).

Molecular similarity of approved drugs

Molecular similarity between chemical compounds is a fundamental and pervasive concept in medicinal chemistry. Although similarity assessment is complicated due to its ambiguous and subjective nature[3], similarity coefficients based on fingerprints (bit or numerical string representations of molecular structure) provide a convenient way to compute molecular similarity on a large scale. We have used the Tanimoto similarity based on MACCS fingerprints (Tc)[4] to assess the similarity of any pair of drugs from our dataset. The ambiguity of the similarity

concept makes it difficult to define a threshold value that indicates a statistically significant level of similarity between two chemical compounds. We implemented a statistical analysis similar to that described by Maggiora and co-workers[3], to compute the threshold Tc for our multi-target drugs dataset.

To define a statistically significant threshold Tc we calculated the Tanimoto similarity between each drug pair combination from a large set of approved drugs in ChEMBL. Supplementary Fig. S2 reports the threshold Tc values as a function of the significance level in terms of p-values. We chose a Tc cut-off of 0.65 (p-val = 0.005) as the threshold to define that two drugs are similar. Then, from our significance analysis and according to[3] the probability that two randomly chosen molecules have a Tc value 0.65 or higher is less than 0.5%.

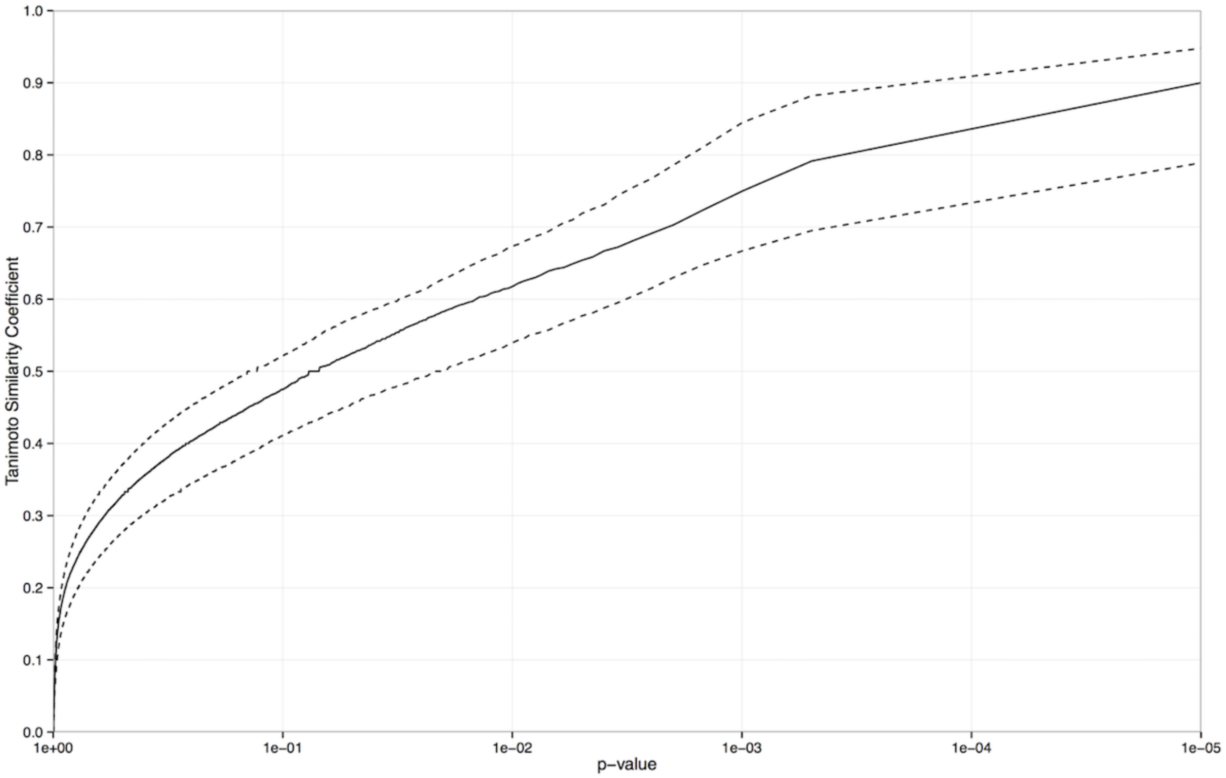

Supplementary Fig. S2. Threshold Tanimoto similarity of approved drugs. Median (solid line) and first and third quartile (dashed lines) of the cumulative distribution function derived from 2015 sampled distributions.

Drug neighbourhoods in the protein functional network

We have shown that the relatives of druggable CATH-FunFams and drug targets have higher kernel similarity than random proteins, meaning that they agglomerate in the protein functional network establishing drug neighbourhoods. Based on the work by Menche et al.[5] we demonstrated that this effect is a property of drug targets and druggable CATH-FunFams rather than caused by the characteristics of the kernel derived from the STRING matrix.

Menche et al. studied the segregation of disease proteins in the same network neighborhoods considering the incompleteness of the interactome and developed a

method to detect network neighbourhoods related to diseases. The situation of network incompleteness described by Menche et al. is pertinent in our study as illustrated in Supplementary Fig. S3. The ideal scenario of a complete network (i.e. a network where all the interactions are known and all the targets of a drug known) leads to easily detecting clusters of drug targets: if we consider a drug d with N_d targets, we would obtain a connected subnetwork of size N_d. However, we always work with incomplete networks and with unknown drug targets. Therefore, clusters of drugs targets might appear as connected subnetworks of size $n < N_d$ close to each other in the network.

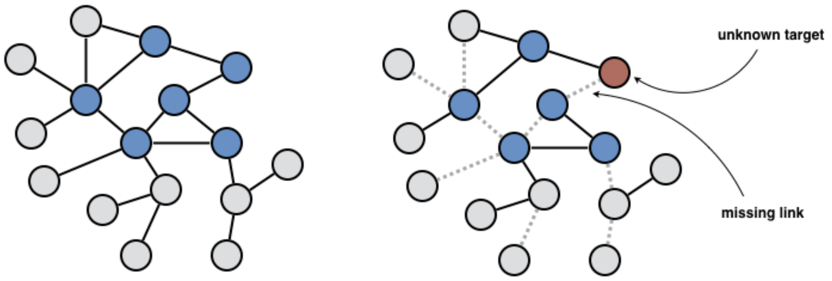

Supplementary Figure S3. Consequence of the network incompleteness and missing targets. The left panel shows the ideal situation of network completeness and where all the targets of a drug d (blue nodes) are known. The targets of d are easily detected as a connected subnetwork of size 6. In the real situation depicted in the right panel, there are only five known targets for d and some missing interactions, the targets of d appear as two small clusters of sizes 2 and 3.

We applied a cut-off of 0.8 on the matrix of STRING combined scores to generate a protein functional network containing high confidence functional interactions among human proteins. We implemented the two measures developed by Menche et al. to quantify the tendency of drug targets to agglomerate in the same

neighbourhoods of this protein functional network: the relative module size, $S = \frac{n_d}{N_d}$, where n_d is the number of targets of the drug d that forms a connected subgraph in the protein functional network and N_d is the total number of targets of d; and the distribution of shortest distances between drug targets, d_s. The relative module size is very sensitive to network incompleteness, as missing links or drug targets in the network may destroy the connected subnetwork and leave the drug targets isolated. d_s is defined for each of the N_d drug targets as the shortest distance to the next closest target of the same drug. Therefore, for each drug we obtain a distribution $P(d_s)$ of N_d data points which average value $\langle d_s \rangle$ can be seen as the diameter of the drug in the network.

We compared the observed drug modules with random models. For each drug with N_d targets we select 1000 random sets of N_d proteins and compute a distribution of 1000 random module sizes S_{rand} and a corresponding distribution of 1000 random $\langle d_s \rangle$ values for which we used its average. As we can see in Supplementary Fig. S4 almost all drug targets are located within 2 links to another target in the network, and ca. 25% of them are directly connected forming close neighbourhoods –the median drug network diameter for drug targets is 1.3 (IQR = 1), whereas for random proteins is 2.8 (IQR = 0.5). Drug targets tend to form larger modules than random sets of proteins. The random sets of proteins are almost never fully connected, usually one third of them form a module, whereas ca. 40% of the observable drug target modules contain more than half of the targets. However, we observe the important effect of network incompleteness as only ca. 15% of the drugs have their targets forming fully connected modules.

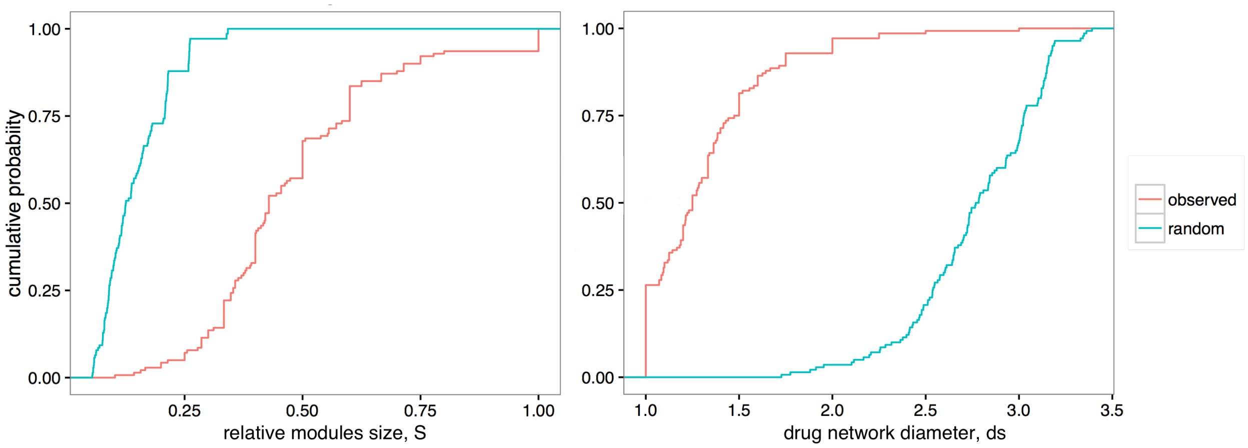

Supplementary Figure S4. Drug modules and drug diameter in the protein functional network. Cumulative distribution function of the drug relative module sizes (left) and the drug network diameters (right) for drug targets and random proteins in the protein functional network.

References

1. Hopkins, A. L. & Groom, C. R. The druggable genome. *Nat Rev Drug Discov* **1,** 727–730 (2002).
2. Kuhn, M., Letunic, I., Jensen, L. J. & Bork, P. The SIDER database of drugs and side effects. *Nucleic Acids Res* **44,** D1075–9 (2016).
3. Maggiora, G., Vogt, M., Stumpfe, D. & Bajorath, J. Molecular similarity in medicinal chemistry. *J Med Chem* **57,** 3186–3204 (2014).
4. Rogers, D. & Hahn, M. Extended-connectivity fingerprints. *J Chem Inf Model* **50,** 742–754 (2010).
5. Menche, J. *et al.* Uncovering disease-disease relationships through the incomplete interactome. *Science* **347,** 1257601–1257601 (2015).
